# Clinical and histopathological features of lentigo maligna and lentigo maligna melanoma: a retrospective analysis in Korea

**DOI:** 10.3389/fmed.2023.1249796

**Published:** 2024-01-05

**Authors:** Chanyong Park, Dong Hyo Kim, Keunyoung Hur, Je-Ho Mun

**Affiliations:** ^1^Seoul National University College of Medicine, Seoul, Republic of Korea; ^2^Department of Dermatology, Seoul National University College of Medicine, Seoul, Republic of Korea; ^3^Department of Dermatology, Seoul National University Hospital, Seoul, Republic of Korea

**Keywords:** Hutchinson’s melanoma, freckle, lentigo maligna, lentigo maligna melanoma, melanoma, pathology, pigmented skin lesions

## Abstract

**Introduction:**

Lentigo maligna (LM) and lentigo maligna melanoma (LMM) are rare in Asian countries. The histopathological diagnosis of LM is often challenging, and misdiagnosis is common. Although histopathologic features of LM/LMM are known, statistical analysis of them were scarcely reported. In this study, we aimed to investigate the histopathological characteristics of LM/LMM in Korean patients and identify key histopathological clues distinguishing LM from benign lentigo.

**Methods:**

We performed a retrospective study of the clinical and histopathological features of patients diagnosed with LM/LMM at our center between 2011 and 2022. We assessed the histopathological features in each case based on 16 pathological criteria according to previous literature. Pathologically confirmed cases of benign lentigo were analyzed for comparison.

**Results:**

Twenty-one patients (10 with LM and 11 with LMM) were analyzed. Several statistically significant difference existed between the features of LM and benign lentigo (*N* = 10), including asymmetry of overall structure (*p* < 0.001), cytologic atypia (*p* < 0.001), predominant single-cell proliferation (*p* < 0.001), melanocytic nests (*p* = 0.033), melanocytes forming rows (*p* = 0.003), pagetoid spread of melanocytes (*p* < 0.001), and hair follicle invasion by atypical melanocytes (*p* < 0.001). Degree of solar elastosis was more severe in group “Age ≥ 60” (*p* = 0.015), and group “Diameter ≥ 20 mm” (*p* = 0.043). Presence of elongated rete ridges were less common in the older than 60 age group (*p* = 0.015) and group “Diameter ≥ 20 mm.” Invasion was associated with mitosis (*p* = 0.001, OR 49.285), multinucleated cells (*p* = 0.035, OR 17.769), and degree of lymphocyte infiltration (*p* = 0.004).

**Conclusion:**

This study investigated the clinical and histopathologic characteristics of LM and LMM in Koreans. Although histopathological diagnosis is challenging, especially in the early stages of LM, our data showed essential histopathological changes in architectural, cytological, and dermal patterns. Considering the potential aggressiveness of LM/LMM, it is essential to recognize its histopathological features and provide timely management.

## 1 Introduction

Lentigo maligna/lentigo maligna melanoma (LM/LMM) is the most common facial melanoma subtype (1). There are significant racial differences in incidence of LM/LMM. According to one study reporting racial differences of epidemiology of melanoma subtypes, age-adjusted incidence of LMM in non-Hispanic white population was 1.87 (1.83–1.90) per 100,000 person-years, while that of Asians/pacific islanders was 0.06 (0.05–0.08) per 100,000 person-years (2).

To the best of our knowledge, there have been few reports on the histopathological features of LM/LMM in Asian patients. In this study, we investigated the clinical and histopathological characteristics of LM and LMM in Korean patients at our center. In addition, we explored the key histopathological clues for the differential diagnosis of LM/LMM in the early stages by comparing LM and benign lentigo.

## 2 Materials and methods

We retrospectively analyzed the clinical and histopathological features of patients diagnosed with LM/LMM at Seoul National University Hospital between 2011 and 2022. Patients with facial and scalp LM/LMM and available histopathological findings were included. The assessed demographic and clinical factors included sex, age at onset, disease duration, lesion location, lesion multiplicity, diameter, depth, presence or absence of metastasis, sentinel lymph node biopsy, clinical impression, presence or absence of symptoms, presence or absence of previous biopsy/laser therapy, previous history of skin cancer, underlying diseases, operation date, and last follow-up date.

We assessed the histopathological features in each case based on 16 pathological criteria according to previous literature (Table 1) (3–5). They consist of one structural change (asymmetry of overall architecture), three cytologic changes (atypical melanocytes, multinucleated melanocytes, and mitosis), seven epidermal patterns (predominant single melanocyte proliferation, melanocytic nests, melanocyte-forming rows, pagetoid spread of melanocytes, hair follicle invasion of atypical melanocytes, sub-epidermal cleft, and the presence of elongated rete ridges), and five dermal changes (dermal lymphocyte infiltration, dermal invasion of atypical melanocytes, solar elastosis, dermal melanophages, and vascular proliferation). We graded the dermal invasion of atypical melanocytes and solar elastosis into three levels (grades 1–3). All the specimens were evaluated by two authors (CP and JHM). Disagreements were resolved by a consensus meeting involving other evaluators (DHK and KH) based on the above definitions in Table 1. Cases of histopathologically confirmed benign lentigo on the face acquired from our database were included as a control group to compare the histological features with those of LM. This study was approved by the Institutional Review Board of the Seoul National University Hospital.

**Table 1:** Definitions of histopathologic variables included in the investigation.

Histopathologic variables	Definition
Structure	
Asymmetry of overall architecture	Defined as present when a central line divided the lesion into two parts that looked different in shape, in thickness, or in number and position of dermal cells, absent only when the lesion appeared perfectly symmetrical
Cytologic change	
Atypical melanocytes	Atypia defined as melanocytic nuclei enlarged (more than keratinocytic ones), variable in size and in shape, hyperchromatic, with eosinophilic or amphophilic nucleoli
Multinucleated melanocytes Mitosis of melanocytes( ≥ 1/10HPF)	Presence of melanocytes that containing more than 1 nuclei (2–3 or more) Presence of melanocytes cell division – considered present when observed more than 1 in 10 HPF
Epidermal change	
Predominant single melanocytes proliferation	Epidermal melanocytes disposed as solitary units predominating over melanocytic nests in some high power fields
Melanocytic nests	Intraepidermal melanocytes were defined as arranged in nests when they formed clusters of five or more cells no matter where they were located (within the basal epidermis or in higher layers of the epidermis)
Pagetoid spread of melanocytes	Melanocytes scattered in the epidermis and in the follicular epithelium in a pattern similar to that of Paget disease
Hair follicle invasion of atypical melanocytes Presence of bulb-like elongated rete ridges	Presence of hair follicle involvement by atypical melanocytes Presence of bulb-like shaped, downward thickening of rete ridges
Dermal change	
Dermal lymphocyte infiltration Degree of lymphocyte infiltration	Defined as present when dermal lymphocytic infiltrate was evident underlying and/or in the context of the lesion Degree of lymphocyte infiltration in dermis. 0 = absent, 1 = mild, 2 = moderate, 3 = severe
Dermal invasion of atypical melanocytes	Invasion of dermis by atypical melanocytes
Presence of solar elastosis Degree of solar elastosis	Presence of elastotic fibers in the normal skin surrounding the melanoma Degree of solar elastosis. 0 = absent, 1 = mild, 2 = moderate, 3 = severe
Dermal Melanophages	Presence of histiocytes with phagocytosed melanin
Vascular proliferation	Presence of (significant) proliferation of vessels

Continuous variables are presented as means ± standard deviation, and categorical variables are presented as frequencies with percentages. Statistical analyzes were conducted using SPSS Statistics 26 software (IBM Corp., Armonk, NY, United States). Fisher’s exact test and the Mann–Whitney U test were used to determine statistically meaningful correlations between demographic and histopathological variables. Statistical significance was set at *p* < 0.05. Odds ratios were calculated using 2 × 2 tables and Haldane’s correction was applied for cells with zero (6).

## 3 Results

### 3.1 Demographics and clinical characteristics of patients with LM/LMM

A total of 21 patients (12 women [57.1%]) with LM/LMM were included in this study (Table 2). Mean age at diagnosis was 68.1 ± 10.63 (range, 49–84). Mean duration of the disease was 8.1 ± 5.26 years (range, 1–20). The most frequent location of the lesions was the cheek in 13 patients (61.9%). Twenty (95.2%) patients had a single lesion, whereas one patient with xeroderma pigmentosum presented with multiple lesions. One patient (4.7%) experienced recurrence after surgery and 20 patients were diagnosed first.

**Table 2:** Patient demographics (*N* = 21).

Characteristic	Value
Age at onset (years, SD)	60, 12.23
Sex, *n* (%)	
Female	12 (57.1%)
Male	9 (42.9%)
Disease duration (years, SD)	8.1, 5.26
Locations, *n*(%)	
Forehead and temple	5 (23.8%)
Nose	1 (4.7%)
Cheek	13 (61.9%)
Lower cutaneous lip	1 (4.7%)
Mucosal lip	2 (9.5%)
Eyebrow	1 (4.7%)
Ear	1 (4.7%)
Lesion number, *n* (%)	
Single	20 (95.2%)
Multiple	1 (4.7%)
Diameter (mm, SD)	24.95, 13.69
Presence/absence of invasion	
*In situ*	10 (47.6%)
Invasive	11 (52.3%)
Depth (mm, SD)	4.23, 5.02
Metastasis, *n* (%)	
No metastasis	19 (90.4%)
Metastasis present	2 (9.5%)
Misdiagnosed as benign lesion a previous biopsy	8 (38%)
Yes	6
No	2
Symptoms, *n* (%)	
Present	3 (14.2%)
None	17 (80.9%)
SLN biopsy, *n* (%)	
Yes	5 (23.8%)
No	16 (76.1%)
Previous Laser therapy, *n* (%)	
Yes	8 (38%)
No	13 (61.9%)
Previous skin cancer, *n* (%)	
Yes	0 (0%)
No	21 (100%)
Underlying diseases, *n* (%)	
Yes	12 (57.1%)
No	9 (42.9%)

SD, standard deviation; SLN, sentinel lymph node.

Mean lesion diameter was 24.95 ± 13.69 mm (range, 5–60). Ten (47.6%) patients had *in situ* melanoma (LM), and 11 (52.4%) had invasive melanoma (LMM). Mean Breslow thickness of the invasive cases was 4.23 ± 5.02 mm (range, 0.3–16). The majority of patients were asymptomatic, while three (14.2%) patients had symptoms such as itching, bleeding, or pain. Notably, eight (38%) patients had a history of misdiagnosis as a benign lesion. Among them, six (75.0%) had undergone skin biopsy at local dermatology clinics but were diagnosed with benign lesions, including junctional nevi or lentigo. The patient had no history of skin cancer. Twenty (95.2%) patients were treated with surgical excision, and one patient (4.8%) was successfully treated with topical imiquimod. Among the patients who underwent surgical treatment, one (5.0%) experienced recurrence. Metastasis was found in two (9.5%) cases. Mean follow-up period was 33.88 ± 21.48 months (range, 2.13 ~ 71.86).

### 3.2 Histopathology features of LM/LMM

Asymmetry of the overall architecture was observed in all the cases (100%). Atypical melanocytes, multinucleated melanocytes, and mitosis were observed in 21 (100%), five (23.8%), and 14 (66.7%) cases, respectively. For epidermal changes, all specimens had a predominant single melanocyte proliferation pattern, while hair follicle invasion of atypical melanocytes, pagetoid spread of melanocytes, melanocyte-forming rows, melanocytic nests, presence of bulb-like elongated rete ridges, and subepidermal clefts were found in 20 (95.2%), 20 (95.2%), 14 (66.7%), 13 (61.9%), 11 (52.3%), and 11 cases (52.3%), respectively. Lymphocyte infiltration was observed in all the cases. When the density of lymphocyte infiltration was graded from 1 to 3 (1 = mild, 2 = moderate, and 3 = severe), grades 1, 2, and 3 were observed in nine (42.8%), ten (47.6%), and two (9.5%) cases, respectively. Dermal invasion of atypical melanocytes was observed in 11 specimens (52.3%). All specimens exhibited solar elastosis, which was graded from 1 to 3 (1 = mild, 2 = moderate, and 3 = severe). Among the 21 specimens, nine were grade 1, eight were grade 2, and four were grade 3. Nineteen (90.4%) patients had dermal melanophages and nine (42.8%) showed vascular proliferation (Table 3).

**Table 3:** Histopathologic data, *n* (%) (*N* = 21).

Histopathologic features	LM (*N* = 10)	LMM (*N* = 11)	*p* value	Odds ratio
*Structure*				
Asymmetry of overall architecture	10 (100%)	11 (100%)	–	1.095
*Cytologic change*				
Atypical melanocytes	10 (100%)	11 (100%)	–	1.095
Multinucleated melanocytes	0	5 (45.5%)	0.035	17.769
Mitosis ( ≥ 1/10HPF)	3 (30%)	11 (100%)	0.001	49.285
*Epidermal change*				
Predominant single melanocyte proliferation	10 (100%)	11 (100%)	–	1.095
Melanocytic nests	5 (50%)	8 (72.7%)	0.387	2.667
Melanocytes forming rows	7 (70%)	7 (63.6%)	1.000	0.75
Pagetoid spread of melanocytes	9 (90%)	11 (100%)	0.476	3.631
Hair follicle invasion of atypical melanocytes	9 (90%)	11(100%)	0.476	3.631
Subepidermal cleft	3 (30%)	8(72.7%)	0.086	6.222
Presence of bulb-like elongated rete ridges	5 (50%)	7(63.6%)	0.670	1.75
*Dermal change*				
Dermal lymphocytes infiltration	10 (100%)	11 (100%)	–	1.095
Lymphocyte density (1, 2, 3)			0.004	
1	8 (80%)	1 (9%)		
2	2 (20%)	8 (72.7%)		
3	0	2 (18.1%)		
Presence of solar elastosis	10 (100%)	11 (100%)	–	1.095
Degree of solar elastosis (1,2,3)			0.376	
1	3 (30%)	6 (54.5%)		
2	4 (40%)	4 (36.3%)		
3	3 (30%)	1 (9%)		
Dermal melanophages	8 (80%)	11 (100%)	0.214	6.764
Vascular proliferation	2 (20%)	7 (63.6%)	0.080	7

LM, lentigo maligna; LMM, lentigo maligna melanoma.

To identify key histopathologically distinguishing points in the early stages of LMM, we further compared the clinical and histopathological features of benign lentigo (*N* = 10) with those of LM (N = 10; Tables 4, 5). Several histopathological features were statistically relevant, including asymmetry of the overall structure (*p* < 0.001, odds ratio [OR] 133), cytologic atypia (*p* < 0.001, OR 441), predominant single-cell proliferation (*p* < 0.001, OR 441), melanocytic nests (*p* = 0.033, OR 21), melanocyte-forming rows (*p* = 0.003, OR 45), pagetoid spread of melanocytes (*p* < 0.001, OR 133), and hair follicle invasion by atypical melanocytes (*p* < 0.001, OR 133).

**Table 4:** Demographics and clinical features between patients with lentigo and LM (*N* = 10, each).

Characteristic	Lentigo (*N* = 10)	LM (*N* = 10)
Age at diagnosis(years, SD)	69.5, 9.99	66.77, 12.46
Sex, *n* (%)		
Female	7 (70%)	7 (70%)
Male	3 (30%)	3 (30%)
Disease duration (years, SD)	4.59, 6.77	8, 4.92
Locations, *n* (%)		
Forehead/Temple	3 (30%)	1 (10%)
Nose	3 (30%)	1 (10%)
Cheek	3 (30%)	7 (70%)
Lip	0	1 (10%)
Eyebrow	1 (10%)	0
Diameter (mm, SD)	12.6, 6.41	22.8, 15.71

SD, standard deviation; LM, lentigo maligna.

**Table 5:** Comparison of histopathologic features of lentigo and LM patients, *n* (%) (*N* = 20).

Histopathologic features	Lentigo (*N* = 10)	LM (*N* = 10)	*p* value	Odd ratio
*Structure*				
Asymmetry of overall architecture	1 (10%)	10 (100%)	<0.001	133
*Cytologic change*				
Atypical melanocytes	0	10 (100%)	<0.001	441
Multinucleated melanocytes	0	0	–	
Mitosis ( ≥ 1/10HPF)	0	3 (30%)	0.211	9.8
*Epidermal change*				
Predominant single melanocyte proliferation	0	10 (100%)	<0.001	441
Melanocytic nests	0	5 (50%)	0.033	21
Melanocytes forming rows	0	7 (70%)	0.003	45
Pagetoid spread of melanocytes	0	9 (90%)	<0.001	133
Hair follicle invasion of atypical melanocytes	0	9 (90%)	<0.001	133
Subepidermal cleft	0	3 (30%)	0.211	9.8
Presence of bulb-like elongated rete ridges	9 (90%)	5 (50%)	0.141	0.111
*Dermal change*				
Dermal lymphocytes infiltration	6 (60%)	10 (100%)	0.087	14.538
Lymphocyte density (1, 2, 3)			1.000	
1	5 (50%)	8 (80%)		
2	1 (10%)	2 (20%)		
3	0	0		
Presence of solar elastosis	10 (100%)	10 (100%)		1
Degree of solar elastosis (1,2,3)			0.148	
1	3 (30%)	3 (30%)		
2	7 (70%)	4 (40%)		
3	0	3 (30%)		

LM, lentigo maligna.

Additional analyzes were performed to evaluate the association between the histopathological findings and clinical features, including age, sex, tumor diameter, and presence of invasion. The degree of solar elastosis was more severe in patients aged >60 years (*p* = 0.015), and elongated rete ridges were more common in patients aged <60 years (p = 0.015). In large lesions (diameter > 20 mm), the degree of solar elastosis was more severe (*p* = 0.043), and elongated rete ridges were less common (*p* = 0.005). Mitosis (*p* = 0.001, OR 49.285) and multinucleated cells (*p* = 0.035, OR 17.769) were significantly more common in invasive cases than in *in-situ* melanoma. The degree of lymphocyte infiltration was higher in the invasive disease group than in the noninvasive disease group (*p* = 0.004).

## 4 Discussion

LM refers to melanoma *in situ* arising on chronically sun-damaged skin (7), and the term “LMM” is used to describe the invasive form of LM (8). LM has the potential to progress to LMM, an invasive tumor with aggressive behavior (9). Therefore, recognizing lesions at an early stage is crucial to minimize the risk of metastasis. The accurate diagnosis of LM/LMM is often challenging, particularly in countries where the incidence of LM/LMM is low. In this study, 38% of the patients were initially misdiagnosed with benign lesions, such as junctional nevi or lentigines, despite previous histopathological examination by skin biopsy. This suggests that it is difficult for pathologists to accurately diagnose LM in its early stages. The lack of experience among pathologists in diagnosing LM and unclear pathologic criteria may contribute to diagnostic delay.

Although several histopathological features of LM/LMM are known (10), essential features for differentiation from benign lentigo have rarely been explored. In this study, the comparison between LM and benign lentigo revealed several statistically significant features, including asymmetry of overall structure, cytologic atypia, predominant single-cell proliferation, melanocytic nests, melanocyte-forming rows, pagetoid spread of melanocytes, and hair follicle invasion by atypical melanocytes. The presence of bulb-like, elongated rete ridges is a characteristic feature of solar lentigo. Although it was observed in the majority of lentigo cases (90% [9/10]), 5 LM (50%) and 6 LMM (54.6%) cases had this pattern. This indicates that the presence of bulb-like elongated rete ridges does not exclude a diagnosis of LM or LMM.

Moreno et al. (3) reported a study of 96 patients with LM/LMM and analyzed the relationship between various histological features and the presence of invasive lesions. In their study, the presence of melanocyte rows (*p* = 0.02, OR 11.5), subepidermal cleft (*p* = 0.049, OR 2.8), melanocytic nests (*p* = 0.04, OR 3.0), and a lower degree of solar elastosis (*p* = 0.07, OR 0.4) were associated with invasive lesions. However, in our study, mitosis (*p* = 0.001, OR 49.285), multinucleated cells (*p* = 0.035, OR 17.769), and a severe degree of lymphocyte infiltration (*p* = 0.004) were associated with invasion. However, the presence of melanocyte rows (*p* = 1.000, OR 0.75), subepidermal cleft (*p* = 0.086, OR 6.222), melanocytic nests (*p* = 0.387, OR 2.667), and the degree of solar elastosis were not significantly associated with invasion. This difference between the two studies may have originated from different sample sizes or ethnicities of the patient groups. This suggests the necessity for further studies regarding the differences in LM/LMM between Western and Asian patients.

The presentation of LM or LMM has several differences between Asian and Western patients. Invasive lesions were more common in Korean patients. In addition, they had larger lesions compared to Western patients. In a study that analyzed patients in the United States with histologically confirmed LM (Navarrete-Dechent, Cristian et al.) (11), the mean overall LM clinical diameter was 11.4 mm (SD, 8.3; range, 2–56 mm). In our study, it was 24.95 mm (standard deviation [SD], 13.69; range: 5–60 mm). The ratio of *in situ* lesions (LM) to invasive lesions (LMM) in the Western study was 2.70, while that in our study was 0.91. Our results were consistent with a previous study of clinical and histologic features of 19 Korean patients (12), in which the majority of cases were invasive, as the ratio of LM/LMM was 0.35 (5 *in situ*, 14 invasive). Compared to that study (12), our data showed a shorter mean disease duration, suggesting some improvement in early diagnosis of LM/LMM. Melanoma overdiagnosis has become a significant global concern, including in the United States (13–15). Data indicate that the incidence of melanoma *in situ* is currently 50 times higher than in 1975 (25 vs. 0.5 per 100,000 population), and the incidence of invasive melanoma has also increased from 7.9 to 25.4 per 100,000 population over the same period (13). However, in South Korea, the incidence of melanoma did not show an exponential increase, and the number of invasive melanoma cases appears to be higher than *in situ* cases. According to data from the Korea Central Cancer Registry, the age-standardized incidence rate of cutaneous melanoma has only mildly increased from 0.51 in 1999–2002 to 0.67 in 2011–2014 among men (average annual percentage change [AAPC], 3.0 [95% CI, 0.8 to 5.3]), and from 0.43 in 1999–2002 to 0.60 in 2011–2014 among women (AAPC, 3.5 [95% CI, 2.4 to 4.6]) (16). Considering previous reports and our data, we believe that the diagnosis of LM/LMM is underreported in South Korea. Given the aggressiveness of melanoma and the management challenges, along with the scarring tendency, primarily when it manifests on the face, that often follows surgical treatment, we believe early diagnosis remains an essential component for effective treatment.

The limitations of our study include its retrospective design and small sample size. However, considering the rarity of LM/LMM in Asians, our study analyzed the clinical and histopathological features of the largest number of Korean patients with LM/LMM. As our patient group consisted only of East Asian patients, further studies are necessary to determine whether there are differences between Western and Asian patients with LM and LM/LMM.

In summary, this study analyzed the clinical and histopathologic characteristics of LM in Korean patients. Compared to Western data, the lesion size was larger, and the ratio of the *in situ* stage (LM) to the invasive stage (LMM) was lower in our study. Although histopathological diagnosis is challenging, especially in the early stages of LM, our data showed essential histopathological changes in architectural, cytological, and dermal patterns. Considering the aggressiveness of LM/LMM, it is important to recognize its histopathological features and provide timely management.

## Data availability statement

The original contributions presented in the study are included in the article/supplementary material, further inquiries can be directed to the corresponding author.

## Ethics statement

The studies involving humans were approved by the Institutional Review Board of the Seoul National University Hospital. The studies were conducted in accordance with the local legislation and institutional requirements. The ethics committee/institutional review board waived the requirement of written informed consent for participation from the participants or the participants' legal guardians/next of kin because retrospective study design, minimal risks to subjects, anonymous data.

## Author contributions

CP, DK, and J-HM conceived and designed the study. CP performed the statistical analyzes. CP and J-HM wrote the first draft of the manuscript. All authors contributed to the article and approved the submitted version.
